# The *cis* and *trans* effects of the risk variants of coronary artery disease in the Chr9p21 region

**DOI:** 10.1186/s12920-015-0094-0

**Published:** 2015-05-10

**Authors:** Wei Zhao, Jennifer A Smith, Guangmei Mao, Myriam Fornage, Patricia A Peyser, Yan V Sun, Stephen T Turner, Sharon LR Kardia

**Affiliations:** Department of Epidemiology, University of Michigan, Ann Arbor, MI USA; Institute of Molecular Medicine and Human Genetics Center, University of Texas Health Science Center, Houston, TX USA; Department of Epidemiology, Rollins School of Public Health, Emory University, Atlanta, GA USA; Division of Nephrology and Hypertension, Department of Medicine, Mayo Clinic, Rochester, MN USA

**Keywords:** GENOA, Gene expression, SNP, CAD, Chr9p21

## Abstract

**Background:**

Recent genome-wide association studies (GWAS) have shown that single nucleotide polymorphisms (SNPs) in the Chr9p21 region are associated with coronary artery disease (CAD). Most of the SNPs identified in this region are non-coding SNPs, suggesting that they may influence gene expression by *cis* or *trans* mechanisms to affect disease susceptibility. Since all cells from an individual have the same DNA sequence variations, levels of gene expression in immortalized cell lines can reflect the functional effects of DNA sequence variations that influence or regulate gene expression. The objective of this study is to evaluate the functional consequences of the risk variants in the Chr9p21 region on gene expression.

**Methods:**

We examined the association between the variants in the Chr9p21 region and the transcript-level mRNA expression of the adjacent genes (*cis*) as well as all other genes across the whole genome (*trans*) from transformed beta-lymphocytes in 801 non-Hispanic white participants from The Genetic Epidemiology Network of Arteriopathy (GENOA) study.

**Results:**

We found that the CAD risk variants in the Chr9p21 region were significantly associated with the mRNA expression of the *ANRIL* transcript ENST00000428597 (p = 8.58e-06)*.* Importantly, a few distant transcripts were also found to be associated with the variants in this region, including the well-known CAD risk gene *ABCA1* (p = 1.01e-05). Gene enrichment testing suggests that retinol metabolism, N-Glycan biosynthesis, and TGF signaling pathways may be involved.

**Conclusion:**

These results suggest that the effect of risk variants in the Chr9p21 region on susceptibility to CAD is likely to be mediated through both *cis* and *trans* mechanisms.

**Electronic supplementary material:**

The online version of this article (doi:10.1186/s12920-015-0094-0) contains supplementary material, which is available to authorized users.

## Background

Atherosclerosis is a condition in which plaque builds up within the walls of the coronary arteries that can lead to a decreased supply of oxygen-rich blood to the heart muscle. Over time, the accumulation of plaques within the coronary arteries can weaken the heart muscle and lead to heart failure and arrhythmias. The end result is coronary artery disease (CAD), which is the leading cause of death in the United States and many western countries [1-3]. Genetic epidemiological studies of families and twins have suggested that genetic mechanisms contribute to CAD susceptibility after adjusting for other major non-genetic risk factors [4]. In particular, genome-wide association studies (GWAS) of common SNPs have found that single nucleotide polymorphisms (SNPs) in the Chr9p21 region are associated with CAD in populations with European ancestry [5-10]. This association was later replicated in many other ethnicities, including Asians and Hispanics [11-13]. In addition to CAD, this locus has also been found to be associated with many other cardiovascular traits, including stroke [14], aneurysm [15,16] and peripheral artery disease [17]. This suggests the Chr9p21 region has broad effects on the cardiovascular system.

The Chr9p21 haplotype block is a 113.5-kb region from base pair 22,012,420 to 22,125,915 on chromosome 9 that contains multiple SNPs that are in high linkage disequilibrium [18]. Even though the association between CAD and the variants in the Chr9p21 region is consistent across many different studies and different ethnicities [5-13], the fact that the SNP associated with CAD in GWAS does not fall within gene boundaries makes the interpretation of the association difficult. Moreover, the association between the Chr9p21 region and CAD is independent of any traditional risk factors, such as hypertension or lipid profile [5-7]. All of this evidence suggests that the Chr9p21 region affects atherosclerosis through a novel mechanism that is yet to be discovered.

Genetic variants can affect RNA expression through either *cis or trans* mechanisms or both. In this paper, the *cis* effect refers to the effects of the genetic variants of the Chr9p21 region on the genes within the same region. The effects of the variants on all other genes are referred to as a *trans* effect. The *cis* effect of the Chr9p21 region has been investigated by several studies. There are four genes that are adjacent to the region of chromosome 9p21, which are *CDKN2A, CDKN2B, ANRIL* and *C9orf53. CDKN2A* and *CDKN2B* are cyclin-dependent kinase inhibitors and are known tumor suppressors [19]. *ANRIL,* also known as *CKDN2B-AS,* is a non-coding RNA [20] and *C9orf53* is an open reading frame. The functions of *ANRIL* and *C9orf53* are not clear. Several studies have examined the genetic effect of the identified risk variants in this region on the abundance of mRNA level of these genes in a variety of cells and tissues [21-27]. The majority of these studies found robust associations between *ANRIL* expression and the Chr9p21 genotypes. All of them suggest an important role for *ANRIL* in the susceptibility of CAD. However, all of these studies have examined only a small number of CAD risk SNPs. There is no evidence so far that the identified risk SNPs are the true functional SNPs. Thus, a larger range of survey, including all of the common and rare variants in the region, is needed. Moreover, alternative splicing plays an important role in the gene expression. Investigating transcript level mRNA abundance, as opposed to the whole gene level, would lend insight into understanding the function. Lastly, despite the importance of the Chr9p21 region, there is no study that particularly investigated the *trans* effects of the region, which may play a more proximal role on atherosclerosis than the *cis* effects [28,29].

The Genetic Epidemiology Network of Arteriopathy Study (GENOA), a network in the Family Blood Pressure Program [30], is a community-based study that recruited sibships with at least two adults with clinically diagnosed essential hypertension before age 60. GENOA has genome wide measurement of exon-level gene expression profiles in immortalized beta-lymphocyte cell lines and genome-wide genotypes on about 800 individuals, which provides a unique epidemiological opportunity to evaluate both the *cis* and the *trans* effect of the variation in the Chr9p21 region on gene expression. Immortalized beta-lymphocyte cell lines are not the most proximal tissue for examining the atherosclerotic process, however, they provide an efficient and robust way to examine the effects of DNA sequence variation on variation in gene expression since the environmental conditions of the cells are the same across individuals (i.e. environmental variation is minimized). We also used a SNP-set based association method [31] to examine the effects of all variants in the 9p21 region at once for transcriptome wide gene expression analysis.

## Methods

### Sample

The GENOA study is a community-based study of sibships that aims to identify genes influencing blood pressure [30,32]. Sibships with at least two adults with clinically diagnosed essential hypertension before age 60 were recruited. All other members of the sibship were invited to participate regardless of their hypertension status. All subjects came from the Rochester, MN field center and were non-Hispanic whites of European descent. Among the participants, 801 individuals had both genome-wide SNP data and gene expression data. The GENOA study was approved by the University of Michigan Health Sciences and Behavioral Sciences Institutional Review Board and the Mayo Clinic Institutional Review Board. Each participant gave written informed consent.

### Genotyping and quality control of GWAS SNPs

GENOA participants were first genotyped using the Affymetrix Genome-wide Human SNP Array 6.0 platform. The samples whose genotyping was not successful on the Affymetrix 6.0 platform were re-genotyped using the Illumina Human1M-DUO BeadChip (Illumina, 2010). For each platform, participants were excluded if they had an overall SNP call rate <95% or sex mismatch between genotypic and phenotypic measurement. SNPs were excluded if they had an unknown chromosomal location or a call rate less than 95%. These quality control filters resulted in 668,293 SNPs in 1,386 subjects available from the Affymetrix platform and 893,545 SNPs in 123 subjects from the Illumina platform for analysis.

Principal component analysis was conducted as an additional quality control step to identify and remove samples with outlying genotype profiles. After forty-five outliers were identified and excluded, a total of 1,464 individuals had genotype data available for analysis.

For this analysis, imputation was done using the European reference panel (87 CEU, 98 TSI, 89 GBR, 93 FIN, 66 MXL, 55 PUR) from the 1000 Genome Project. Prior to imputation, SNPs were additionally filtered for minor allele frequency (MAF) ≥ 0.01 and Hardy-Weinberg P-value ≥ 0.001. MACH (v1.0.16) and Minimac (v 4.4.3) were used to impute genotypes of unmeasured markers, up to total of 21,880,364 SNPs. MaCH was used in the pre-phasing stage, and Minimac was used in the imputation stage.

After the above steps, 180 SNPs within the region of Chr9p21 (chr9: 22,012,420-22,125,915 bp) that had imputation quality score R^2^ greater than 0.9 and MAF greater than 0.0025 were selected for analysis.

### Gene expression profiling and quality control

RNA samples from cell lines established from peripheral blood samples by Epstein-Barr virus transformation were extracted using standard protocols. RNA quality was assessed using the Agilent 2100 Bioanalyzer (Agilent Technologies Inc., Foster City, CA) and quantified by spectrophotometry using the Nanodrop ND-1000 (Nanodrop Inc., Wilmington, DE). All RNA samples used in the present study yielded both anA260/A280 absorbance ratio greater than 2.0 and a RNA Integrity Number (RIN) ≥ 8.

One μg of RNA was labeled using the WT Expression labeling assay (Applied Biosystems/ Ambion, Foster City, CA) including the labeling controls from the GeneChip Eukaryotic Poly-A RNA Control Kit (Affymetrix, Santa Clara, CA). Each step of the labeling protocol was monitored using the Agilent 2100 Bioanalyzer or the Nanodrop spectrophotometer, as recommended by the manufacturer. Hybridization buffer, Eukaryotic Hybridization Controls, and OligoB2 controls were added to the cDNA fragments just prior to hybridization to the Affymetrix Human Exon 1.0 ST Array. Hybridization was performed at 45°C for 17 hours. Following hybridization, the chips were washed and stained with a phycoerythrin-strepavidin conjugate and were scanned at the excitation wavelength of 488 nm.

Array quality control was performed using Affymetrix Expression Console™ (v 1.1) at the transcript level using core-level probe sets. All array images passed visual inspection. Hybridization controls were all present with signal increases following concentration. Labeling control signal strengths followed the order Lys < Phe < Thr < Dap. Signal intensity plots were examined for raw and processed data to identify outliers. Summarization QC metrics, including relative log expression (RLE), positive vs. negative ROC AUC and MAD-residual mean, were all within the parameters suggested by Affymetrix.

Raw intensity data were processed using Multiple Exon Array Preprocessing (MEAP) software. The PM-BayesBG method was used for background correction. This was followed by quantile normalization, expression summarization and log_2_ transformation [33]. The expression summarization is multi-dimensional depending on the user defined data type, which includes probeset, exon, spliced variant or gene. The transcript (spliced variant) level summary was used in this study. Annotation version 64 was used for our expression summarization. Exon expression intensities were summarized directly from probe-level expression values and MEAP annotation. Expression summarizations for transcripts were calculated from exon expression by linear algebra based on transcript records in external databases. If the probes targeting an exon did not have strong signals and thus caused low or no expression of the exon, it may have resulted in a negative algebraic solution. The transcript’s expression values were denoted as missing in that case. If a transcript had a missing rate greater than 10%, it was excluded from the analysis.

Principal component analysis on the exon level expression data was used to identify possible outliers, and none were identified. We also verified sex assignment of the samples by examining expression levels of genes on the Y chromosome. Two individuals were excluded due to sex mismatch. There were a total of 875 individuals who had gene expression data. Among them, 801 individuals had both gene expression and SNP data.

### Statistical methods

To evaluate the *cis* effect, the association between each SNP in the Chr9p21 region and the candidate transcripts from the four adjacent genes were tested using linear mixed models with each transcript as the outcome, SNP as the predictor, and age, sex and batch as the covariates. Familial correlation was adjusted by the random effect. SNPs were coded additively. Since there were 8 transcripts and 180 SNPs in total, the Bonferroni corrected p value for each transcript was 3.47e-5. Among those SNPs, we also specifically examined six known CAD risk variants: rs7865618, rs10811650, rs1333040, rs10757274, rs2383206, and rs1333045 [34].

In order to increase our power to detect *trans* effects, SNP-set (Sequence) Kernel Association Test (SKAT) [31] was used to test the association between gene expression and the SNP-set within the Chr9p21 region. SKAT allows different weights to be assigned to different variants according to a hypothesis or existing knowledge. The default weight follows a Beta(1,25) function, which assumes rare causal variants have larger effects, and thus assigns larger weights to rare variants. Beta(1,1) assumes all of the causal variants have similar effect sizes and assigns equal weights to all the variants. Beta(0.5,0.5) produces the Madsen & Browning weights that have been a popular choice, which also assigns weight based on the minor allele frequency (MAF) but does not up-weight rare variants as much as the Beta(1,25) function [35]. In order to assess the effect of both rare variants and common variants, we applied all three weighting schemes to the analysis. To adjust for familial correlation, we implemented a two-step approach. First, the transcript level gene expression was adjusted for familial correlation by a linear mixed model and the random residual was saved. Second, SKAT was used to test the association between the residual from the previous step and all the SNPs within the Chr9p21 region, with batch, age and sex as the covariates. SNPs were coded additively. A total of 35,477 transcripts across the whole genome were analyzed, false discovery rate (FDR) at 10% was applied to adjust for multiple testing. To identify *trans* expression quantitative trait loci (eQTLs) at the SNP level, we followed up each SKAT association that had FDR q < 0.1 with single SNP analysis using all of the SNPs in the Chr9p21 region. The statistical model was the same as the model used for the *cis* effect analysis.

Since the transcripts across the genome are highly correlated, it is often too conservative to simply apply a stringent criterion for association. Thus we also performed gene set enrichment testing for transcripts at least nominally associated with the Chr9p21 region (p-value < 0.01) in the Beta(1,25), Beta(1,1) and Beta(0.5,0.5) SKAT analyses. DAVID [36] was used for gene set enrichment testing, in which the background was set as the total of 35,477 transcripts. The p-value of each gene set was calculated using a modified Fisher exact test.

All statistical analyses were performed with R statistical environment version 2.9.0 from R Project (http://www.r-project.org/). The authors had full access to the data and take responsibility for its integrity. All authors have read and agree to the manuscript as written.

## Results

There were 801 individuals who had both expression profiles and SNP data. There were 337 men and 464 women, and the mean age was 59.2 years. The majority of the participants (75.9%), by study design, had hypertension. There were 82 subjects with a history of coronary artery disease.

The Chr9p21 haplotype block is a 113.5-kb region from base pair 22,012,420 to 22,125,915 on chromosome 9 [18]. The adjacent four genes of this region are *ANRIL, CDKN2A, CDKN2B* and *C9orf53* and the available transcripts are presented in the first column in Table 1. In our data, *ANRIL* has 2 transcripts, *CDKN2A* has 3 transcripts, *CDKN2B* has 2 transcripts and *C9orf53* has 1 transcript. The effect of each of the 180 Chr9p21 SNPs on each transcript was evaluated. The SNP with the strongest association with each transcript is shown in Table 1. The Bonferroni corrected alpha level is 3.47e-5. Among the 8 SNPs shown in Table 1, only the top SNP for the *ANRIL* transcript ENST00000428597 was significantly associated after Bonferroni correction. No other transcripts were significantly associated with any SNPs in the Chr9p21 region.

**Table 1:** **Top SNPs associated with each transcript**

**Transcript ID**	**Top SNP**	**Coded allele**	**Non coded allele**	**Coded allele frequency**	**Parameter estimate**	**SE**	**P-value**
*ANRIL*							
ENST00000428597	rs7865618	A	G	0.569	0.07	0.016	8.58e-06
ENST00000422420	rs1759417	C	T	0.834	-0.08	0.045	0.088
*C9orf53*							
ENST00000441769	rs662463	G	A	0.896	0.07	0.024	0.006
*CDKN2A*							
ENST00000380151	rs647188	T	G	0.891	-0.07	0.055	0.199
ENST00000361570	rs647188	T	G	0.891	-0.07	0.045	0.121
ENST00000304494	rs647188	T	G	0.891	-0.06	0.047	0.209
*CDKN2B*							
ENST00000380142	rs2095144	G	A	0.707	0.03	0.012	0.030
ENST00000276925	rs1333040	T	C	0.605	0.02	0.011	0.070

**Figure 1:**
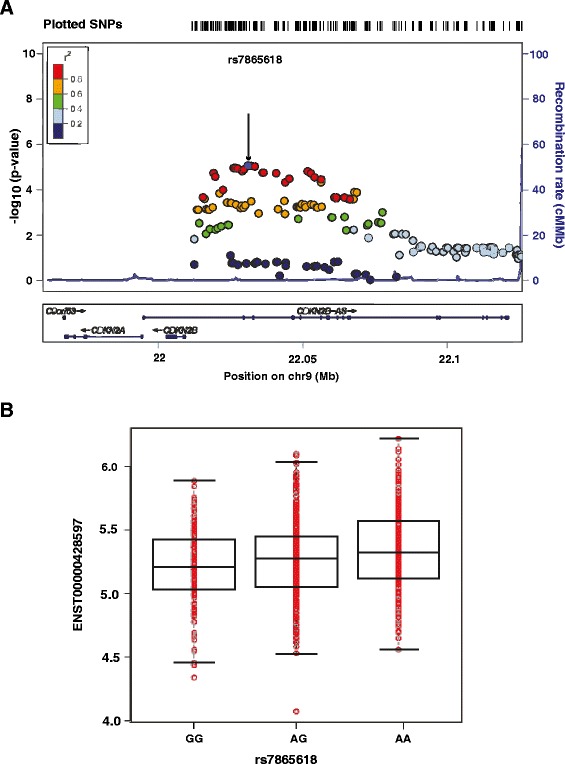
The association between the *ANRIL* transcript ENST00000428597 and the SNPs in the Chr9p21 region. **A**. The relationship between the *ANRIL* transcript ENST00000428597 and the SNPs in the Chr9p21 region. SNPs are plotted by chromosomal position against the –log(p-value) of their association with the *ANRIL* transcript ENST00000428597. The most significant SNP is shown as a purple circle, and indicated by an arrow. The SNPs surrounding the most significant SNP are color-coded to reflect their linkage disequilibrium with this SNP as shown in the inset (taken from pairwise r^2^ values from the 1000 Genomes Project EUR database). Genes and the position of exons, as well as the direction of transcription, are noted below the plots (data from UCSC Genome Browser). **B**. Plot of the mRNA expression of *ANRIL* transcript ENST00000428597 and the top SNP rs7865618. The mRNA expression level (log_2_ scale) is plotted against each genotype and is shown in red. The black boxplot is added on top of the scatterplot to show the median and interquartile range of expression level for each genotype.

Next, we plotted the p value of the association between the *ANRIL* transcript and each SNP against the chromosome position in Figure 1A. There were a total of 18 SNPs that were significantly associated with the mRNA expression of the *ANRIL* transcript ENST00000428597 at the Bonferroni corrected p value. As shown in Figure 1A, almost all the SNPs that were associated with the *ANRIL* transcript are in linkage disequilibrium with the top SNP rs7865618. The mRNA expression level of *ANRIL* transcript ENST00000428597 was plotted against SNP rs7865618 as shown in Figure 1B. One increased copy of A in rs7865618 is associated with a 7% increase in the *ANRIL* transcript ENST00000428597 on the log_2_ scale, which corresponds to a 5% increase on the original scale.

We also specifically examined the association between known CAD risk SNPs in the Chr9p21 region and the *ANRIL* transcript ENST00000428597, and the results are summarized in Table 2. Our top SNP, rs7865618, is included in the group of CAD risk SNPs. Moreover, all of the six candidate SNPs showed a trend of association with this transcript, and three were significantly associated at a nominal level (p = 0.05). Because we know that there is strong linkage disequilibrium in the Chr9p21 region, we examined the correlation among the six candidate SNPs in our dataset and they were all correlated with each other (absolute r ranges from 0.538 to 0.976). For all of the CAD risk SNPs in the Chr9p21 region, the risk variant always increases the mRNA expression of the transcript.

**Table 2:** **The association between** ***ANRIL*** **ENST00000428597 and known CAD risk variants**

**CAD risk SNP**	**Risk allele**	**Coded allele**	**Non coded allele**	**Coded allele frequency**	**Parameter estimate**	**SE**	**P-value**
rs7865618	A	A	G	0.569	0.070	0.0155	8.58e-06
rs10811650	G	G	A	0.490	0.043	0.0154	0.0058
rs1333040	T	T	C	0.605	0.028	0.0158	0.0755
rs10757274	G	G	A	0.490	0.030	0.0152	0.0522
rs2383206	G	G	A	0.504	0.033	0.0153	0.0345
rs1333045	C	C	T	0.543	0.030	0.0158	0.0588

In order to better understand the potential importance of genetic variation in this chromosomal region on the larger metabolic processes underlying the atherosclerotic processes, we used the SKAT method to perform a transcriptome-wide assessment of the Chr9p21 region’s *trans* effect. The top transcripts that were associated with the Chr9p21 region through *trans* effects are listed in Table 3. When the Beta(1,25) weight was used, four transcripts from four genes showed significant association with the Chr9p21 region. They were *DUT* (ENST00000331200), *EIF1AY* (ENST000000361365), *CASP14* (ENST00000427043), and *ABCA1* (ENST000000374736). When the Beta(1,1) and Beta(0.5, 0.5) weighting schemes were used, the top transcript from those two tests was the same for the same gene: *DHRS9* (ENST00000412271). However, this transcript was not significant after controlling for multiple testing. As a comparison, we also provide the SKAT test results for the *ANRIL* transcript (ENST00000428597) that has shown significant association with multiple SNPs in the Chr9p21 region. This transcript was significant in the SKAT test when the weights Beta(1,1) or Beta(0.5,0.5) were applied, but was not significant when the weight Beta(1,25) was applied. As shown in Table 3, the transcript *DHRS9* (ENST00000412271) was more significant than the *ANRIL* transcript, which is known to be associated with the Chr9p21 region. Thus, this gene was also included for the following single SNP analysis.

**Table 3:** **The top transcripts associated with the Chr9p21 region from the SKAT test**

**Transcript**	**N**	**SKAT**	**SKAT**	**SKAT**	**FDR q value**	**Gene**	**Location**
		**Beta(1,25)**	**Beta(1,1)**	**Beta(0.5,0.5)**			
		**P value**	**P value**	**P value**			
Top transcripts when SKAT_Beta(1, 25) was used							
ENST00000331200	800	8.19e-07	0.905	0.416	0.0276	*DUT*	15q21
ENST00000361365	781	1.55e-06	0.762	0.360	0.0276	*EIF1AY*	Yq11
ENST00000427043	796	5.79e-06	0.909	0.490	0.0685	*CASP14*	19p13
ENST00000374736	801	1.01e-05	0.719	0.387	0.0893	*ABCA1*	9q31
Top transcript when SKAT_Beta(1, 1) was used							
ENST00000412271	749	0.608659	3.05e-05	3.38e-05	0.9998	*DHRS9*	2q31
Top transcript when SKAT_Beta(0.5, 0.5) was used							
ENST00000412271	749	0.608659	3.05e-05	3.38e-05	0.9998	*DHRS9*	2q31
*cis* transcript							
ENST00000428597	801	0.47	0.002013	0.001907	1	*ANRIL*	9p21

Next, we looked at the associations between transcripts and single SNPs using the identified top *trans-*effected transcripts and all of the SNPs in the Chr9p21 region. The top SNP for each transcript is listed in Table 4. As shown in Table 4, the top SNPs (rs16923583, rs41386451, rs16905597) for *DUT*, *EIF1AY*, *CASP14* and *ABCA1* were all rare variants (MAF approximately 0.005). Moreover, we found that the top 4 SNPs for all four of these genes were the same rare variants (rs16923583, rs41386451, rs16905597 and rs73650008), although the rank of the associations are different across genes (Additional file 1: Table S1). The top SNP (rs4977756) for the transcript *DHRS9* was a common SNP (MAF = 0.44), which was also correlated with all of the CAD risk SNPs in the Chr9p21 region (absolute r ranges from 0.58 to 0.83). These results are consistent with the underlying assumptions of the three different SAKT weighting schemes that have been used in this study. One common problem for rare variant analysis is that the detected associations are often driven by one or a few samples. That is what we observed in our study as well (Additional file 2: Figure S1). Thus, functional studies are needed to validate our findings.

**Table 4:** **The top SNPs associated with the** ***trans*** **-effected transcripts identified by SKAT**

**Gene**	**Transcript**	**Top SNP**	**Coded allele**	**Non coded allele**	**MAF**	**Beta**	**SE**	**P value**
*DUT*	ENST00000331200	rs16923583	T	A	0.9944	1.07	0.187	2.14e-08
*EIF1AY*	ENST00000361365	rs41386451	A	C	0.9952	1.77	0.377	3.66e-06
*CASP14*	ENST00000427043	rs16923583	T	A	0.9944	1.25	0.236	1.95e-07
*ABCA1*	ENST00000374736	rs16905597	G	A	0.9951	-0.58	0.108	1.65e-07
*DHRS9*	ENST00000412271	rs4977756	A	G	0.5635	0.35	0.089	9.84e-05

To further examine the *trans* effect in multiple tissues and across cohorts, we searched the above identified associations between gene expression levels and Chr9p21 SNPs (SNP rs4977756 with *DHRS9* as well as the 4 rare variants with *ABCA1, CASP14, DUT, EIF1AY*) in two public eQTL databases, GENe Expression VARiation (Genevar) [37] and the Genotype-Tissue Expression project (GTEx) [38]. We preferentially focused on the HapMap3 project in Genevar and heart related tissue in GTEx. At alpha level of 0.05, *DHRS* was found to be associated with rs4977756 in transformed B-lymphoblastoid cells (TBLs) in European Americans (CEU, p = 0.017) and the direction of effect was consistent with the observed effect direction in GENOA*.* However, there was not a significant association between rs4977756 and any heart related tissue in GTEx*.* Only two of the four rare variants, rs16923583 and rs16905597, were available in a small number of non-CEU populations in Genevar, and the associations were not significant. The other two rare variants were not available in either of the databases.

As an alternative, we looked up the association between the six known common Chr9p21 CAD risk SNPs (Table 2) and the genes *DUT*, *EIF1AY*, *CASP14* and *ABCA1.* In the Genevar database, we found that rs10811650 was associated with *ABCA1* expression (*p* = 0.015) in TBLs in CEU and *CASP14* expression (*p* = 0.0017) in an African population (LWK). Also, rs1333040 was associated with *DUT* expression (*p* = 6.0 × 10^-4^) in a separate African population (YRI). Moreover, in the GTEx database, *ABCA1* was associated with four SNPs (rs1333045: p = 0.009; rs2383206: p = 0.002; rs10757274: p = 0.003; rs7865618: p = 0.008) in 74 artery aorta samples. *EIF1AY* was associated with the same set of SNPs in 87 heart left ventricle samples (rs1333045: p = 0.006; rs2383206: p = 0.02; rs10757274: p = 0.005; rs7865618: p = 0.007). This association between Chr9p21 SNPs and gene expression in left ventricular and aorta tissue provide suggestive evidence that the Chr9p21 region has *trans* effects in relevant tissues for several of the genes that we have identified.

To discover the potential biological importance of *trans*-effected genes, we used the KEGG Pathway for gene enrichment analysis. For the top transcripts in Beta(1,25), there was no significantly enriched gene set with p-value < 0.1. However, for the top transcripts in Beta(1,1) and Beta(0.5,0.5), there were two and three gene sets, respectively, that were enriched at a nominal p-value < 0.1. As shown in Table 5, the enriched pathways include retinol metabolism and TGF-β signaling for Beta(1,1), and all of the same pathways plus N-Glycan biosynthesis for Beta(0.5,0.5). The top enriched pathway is retinol metabolism, in which *DHRS9* (ENST00000432060, ENST00000412271), *ADH1* (ENST00000394887), *DHRS4L2* (ENST00000335125), *LRAT* (ENST00000336356) and *CYP26B1* (ENST00000001146) are significantly associated with the Chr9p21 region. One of the two transcripts of *DHRS9* (ENST00000412271) is the most significant *trans*-effect gene in Beta(1,1) and Beta(0.5,0.5).

**Table 5:** **Gene set enrichment testing of KEGG pathway for nominally significant transcripts in Beta(0.5,0.5) and Beta(1,1) (p-value < 0.1)**

	**Term**	**Number of genes**	**P value**	**Enriched transcript**
**Beta(0.5,0.5)**	hsa00830:Retinol metabolism	5	0.016	ENST00000394887, ENST00000001146, ENST00000432060, ENST00000412271, ENST00000336356
	hsa04350:TGF-beta signaling pathway	4	0.056	ENST00000416274, ENST00000224764, ENST00000394092, ENST00000401753
	hsa00510:N-Glycan biosynthesis	3	0.077	ENST00000393487, ENST00000413355, ENST00000250498
**Beta(1,1)**	hsa00830:Retinol metabolism	6	0.002	ENST00000335125, ENST00000394887, ENST00000001146, ENST00000432060, ENST00000412271, ENST00000336356
	hsa04350:TGF-beta signaling pathway	4	0.056	ENST00000416274, ENST00000224764, ENST00000394092, ENST00000401753

## Discussion

Even though the Chromosome 9p21 region has been implicated as a hotspot associated with CAD, it continues to be a challenge to understand the immediate biological and functional effects of variants in that region. Studying the association between genetic variants and gene expression variation is valuable for connecting variation at the DNA sequence level to that at the RNA level. In this study, we used a large family-based cohort where both genotype and gene expression were measured on the same individuals to evaluate both the *cis* and the *trans* effects of the genetic variants in the Chr9p21 region on the RNA expression at the transcript level. It was found that most CAD associated SNPs in the Chr9p21 region were highly associated with the expression of the *ANRIL* transcript ENST00000428597. In addition, we found that the Chr9p21 region was also associated with gene expression of a few distant functional genes and pathways. These results suggest that the genetic effect of risk variants in Chr9p21 region on susceptibility to CAD is likely to be mediated through both *cis* and *trans* effects.

There have been numerous studies looking at the local functional impact of the Chr9p21 variants on gene expression in a variety of tissues and cells [21-27]. All of these studies consistently showed the CAD risk variants in the Chr9p21 region were associated with *ANRIL* expression and have very little effect on the other local transcripts. For example, earlier studies found that a CAD risk variant was associated with reduced expression levels of *ANRIL* in purified peripheral blood T-cells, whole blood cells and vascular smooth muscle cells [22,24,27]. However, these studies only examined *ANRIL* expression at the whole gene level and did not take alternative splicing into account, which could have masked the true effect. Our study examined the mRNA expression at the transcript level and found that SNP rs7865618, which is a CAD risk variant [6,34], was associated with the gene expression of the *ANRIL* long transcript ENST00000428597. The risk variant A increased the ENST00000428597 expression in our study. In addition to SNP rs7865618, we also examined five other CAD-associated SNPs and found that two of them were associated with *ANRIL* expression at a nominal level (p value < 0.05) and the other three also showed a non-significant trend for association. The directions of the effects were the same for all of the CAD risk variants we examined in our study. This replicated the previous report that the same transcript was increased in the carriers of the risk haplotype in peripheral blood mononuclear cells and whole blood cells [25]. However, Jarinova et al. also specifically investigated the relationship between CAD risk variants and different splicing variants of *ANRIL* and reported that the CAD risk variant was associated with a decreased amount of the same long variant in whole blood cells [21]. Congrains et al. did not observe a strong association between the risk variants and the *ANRIL* long transcript in vascular smooth muscle cells [26].

One possible reason that the directions of effects are inconsistent is that these studies were performed in different tissues and have used different assays to target different exons. The transcript ENST00000428597 was the longest transcript of the gene *ANRIL*, which includes all 19 exons. Our study used the Affymetrix Human Exon 1.0 ST Array, which was designed to measure every exon in the gene and then summarize them into transcript level measurements based on known transcript composition. The other three studies used quantitative reverse transcription polymerase chain reaction assays with different probes in their studies (Holdt et al. targeted exon 18-19, Jarinova et al. targeted exon 15-16, Congrains targeted exon 17-18) [21,25,26]. As shown by Folkersen et al., *ANRIL* has many novel transcript variants depending by the tissue type [23]. Thus it is possible that different transcripts were measured in different studies or the same transcript behaved differently in different tissues and environments. A recent functional study suggests that the CAD risk variants promote *ANRIL* expression by disrupting STAT1 binding [39]. Nonetheless, taken together these results along with the fact that *ANRIL* is expressed in cells and tissues that are affected by atherosclerosis [27,40] and are correlated with atherosclerosis severity [25,26], they suggest that *ANRIL* plays an important role in mediating the genetic effect of the Chr9p21 region on the susceptibility of atherosclerosis.

There are several ways that the noncoding RNA *ANRIL* can alter expression of associated protein coding genes, which include RNA interference, gene silencing, chromatin remodeling, and DNA methylation [41]. Recent studies on animals found that *ANRIL* knockdowns showed a substantial reduction of cellular proliferation, which is known to play an important role in atherosclerosis [42,43]. The important role of *ANRIL* on cell growth has also been observed in human vascular smooth muscle cells [26,27]. Other evidence suggests that *ANRIL* may also affect various genes by *trans* effect [44-46]. Further studies are needed to clarify the mechanism involved.

The present study is the first one that has focused on the *trans* effect of the variants in the Chr9p21 region across the whole genome. SKAT is a powerful SNP set association test, which requires very few assumptions [31]. We used this test to screen the whole genome and found that five transcripts from five genes were at least nominally associated with the Chr9p21 region. We also examined the association between the transcripts identified via *trans* analysis and single SNPs within the Chr9p21 region, and we found several *trans*-eQTL SNPs, including both rare and common variants. The common *trans*-eQTL SNP rs4977756 was in the same linkage disequilibrium block with known CAD risk variants, and its association with *DHRS9* was supported by the same signal observed from the same tissue in the HapMap CEU sample. Although the other rare *trans*-eQTL SNPs were not available in the public database, we did examine the relationship between gene expression in our identified *trans*-effected genes and common CAD risk SNPs in the Chr9p21 region, and we found quite a few *trans*-eQTL SNPs. This provides evidence that the Chr9p21 region is indeed associated with the top *trans*-effected genes identified by SKAT. Nonetheless, studies with rare variants and larger sample size are needed to validate our findings.

Among the *trans*-effected genes identified by SKAT, the most well-known gene is *ABCA1*, which is also located on chromosome 9. It encodes a membrane-associated protein that is a member of the superfamily of ATP-binding cassette (ABC) transporters. The ABC protein transports various molecules across membranes and has been shown to be an important regulator for cholesterol efflux from peripheral cells, and mutations in *ABCA1* contribute to low HDL [47-49]. It is known that low HDL cholesterol is an independent risk factor for CAD [50]. Given its role in lowering HDL cholesterol, loss of function mutations in *ABCA1* would increase CAD risk. This supposition has been supported by the observation that the mutations in *ABCA1* are associated with coronary artery calcification, a subclinical trait of atherosclerosis [51] as well as CAD [52]. The function of *ABCA1* in the development of atherosclerosis has also been confirmed in animals. *ABCA1* knockout mice develop larger atherosclerotic lesions, and overexpression of *ABCA1* inhibits the progression of atherosclerotic lesions [53-55]. It was suggested that *ABCA1* protects against atherosclerosis by removing cholesterol from macrophages in the artery wall and therefore prevents the formation of foam cells, which are critical for the formation of atherosclerotic plaque [56,57]. Though the Chr9p21 region has been implicated in the etiology of atherosclerosis for a long time, this is the first time that we have observed an association between this chromosomal region and a functional gene for atherosclerosis. Understanding the cellular mechanism underlying this association is a very important next step to advancing our understanding of the relationship between Chr9p21 and atherosclerosis.

The four other genes identified as significantly associated with the Chr9p21 region are *CASP14*, *EIF1AY*, *DUT* and *DHRS9. CASP14* plays a central role in the execution phase of cell apoptosis and is overexpressed in tumors [58]. *EIF1AY* is on the Y chromosome and is overexpressed in men who have experienced a stroke [59]. *DUT* encodes an essential enzyme of nucleotide metabolism, which is vital to DNA replication [60]. *DHRS9* encodes an enzyme that is involved in the metabolism of retinol [61]. Very little is known about these genes in terms of their roles in CAD, so future studies will be necessary.

Since the transcripts across the genome are highly correlated, applying a stringent p-value cutoff to *trans*-effect analyses may be too conservative. Gene enrichment testing is a powerful way to reveal potential influential transcripts and pathways. According to the gene set analysis, the *trans*-regulated genes predicted by Beta(1,1) and Beta(0.5,0.5) are enriched in several pathways. Retinol metabolism, the top pathway, has shown to have important effects on atherogenesis, endothelial function, vascular calcification and lipid metabolism [62]. It is also correlated with alcoholic liver disease in which alcoholic patients display hepatic total retinol levels that are progressively lower with increasing severity of liver disease [63]. Notably, *DHRS9* has two transcripts within the nominally significant transcripts identified by the SKAT test, increasing the credibility that this gene shows a true *trans*-effect. *ADH1*, one class of alcohol dehydrogenase, catalyzes the oxidation of alcohols to aldehydes and also metabolizes retinol, hydroxysteroids, and lipid peroxidation products. It affects cardiovascular function by regulating alcohol digestion as well as via a direct effect on the vasculature [64]. Increased expression of *CYP26B1* may aggravate atherosclerosis [65].

N-glycan biosynthesis is another pathway that was enriched in the gene set analysis. N-glycans located at the surface of the endothelium are responsible for interactions with monocytes. A previous study has demonstrated that N-glycans are increased on the endothelial cell surface at sites of early human lesion development and are effectors of monocyte adhesion during atherogenesis [66]. The *MAN1A2* gene, which was in this gene set, was reported to be down-regulated by TNFα and is part of an inflammatory mechanism in the pathological process of atherosclerosis.

Activation of the TGF-β pathway is well recognized as the most important feature in the pathogenesis of cardiac remodeling and fibrosis. It also plays a crucial role in the regulation of vascular function. A series of papers reveals the effects of TGF-β signals in angiogenesis and atherosclerosis [67]. *BMPR1A* and *SMAD1* in our gene set are two core components in BMP pathway, which has shown to be related to pulmonary arterial hypertension and vascular calcification. These results all suggest that the risk variants of CAD in the Chr9p21 region may exert the *trans* effect by modulating the expression of genes responsible for critical functions involved in CAD.

There are a few limitations of the current study. First, as there is a considerable amount of gene-expression variation among populations [68], these results may not be applicable to other populations. For example, although the GENOA sample was a community-based sample of sibships selected on hypertension criteria, the frequency of effects could be different from other populations or a representative population-based sample of unrelated individuals. However, our previous contributions to studies in this region do not indicate significant allele frequency differences from other population-based epidemiological samples of unrelated non-Hispanic whites [69]. Second, transformed beta-lymphocytes were used in this study, and thus our results cannot be directly applied to other tissues in the *in vivo* environment. Third, our sample size is relatively small for a transcriptome-wide association analysis.

Given the increased availability of both SNP data and gene expression profiles in epidemiologic samples, investigating both *cis* and *trans* effects to characterize potentially functional elements in genomic regions identified by GWAS is an important area of research. Considering the limitations of our particular study, future epidemiologic studies with larger sample sizes and more ethnically-diverse populations are needed to replicate our findings. In addition, gene expression analyses with rare variants that are conducted in heart related tissue are another important next step for replication. However, whole genome scale transcriptome association analysis only serves as a screening procedure to provide potential targets for the research community. Thus, functional studies are also needed to examine how genetic variation in the Chr9p21 region plays a role in the etiology of CAD.

## Conclusions

In conclusion, we used a novel SNP-set based method to specifically examine the genetic effect of the CAD risk variants of the Chr9p21 region on the transcript level gene expression across the whole genome. We replicated the previous finding that the *ANRIL* long variant is highly associated with the CAD risk variants. Moreover, we provide the first evidence that variation in the Chr9p21 region is associated with important functional genes and pathways, which play an important role in the progression of atherosclerosis. Taken together, this suggests that DNA sequence variation in the Chr9p21 region may affect CAD through both *cis* and *trans* mechanisms.

## Additional files

Additional file 1: Table S1. Four SNPs in the Chr9p21 region associated with all four top transcripts identified by SKAT_Beta(1,25). Additional file 2: Figure S1. The mRNA expression (log_2_ scale) of the top 5 *trans-*effected transcripts (identified by SKAT) was plotted against each genotypes for the corresponding top SNPs (shown in red). Boxplot was added on top of each scatterplot to show the median and interquartile range of the expression level for each genotypes (shown in black). The five plots are for gene A: *DUT* (ENST00000331200), B: *EIF1AY* (ENST00000361365), C: *CASP14* (ENST00000427043), D: *ABCA1* (ENST00000374736) and E: *DHRS9* (ENST00000412271).

## Abbreviations

GWAS: Genome-wide association studies
GENOA: The Genetic Epidemiology Network of Arteriopathy
SNP: Single nucleotide polymorphism
CAD: Coronary artery disease
eQTL: Expression quantitative trait loci
