# Supplementary material for: The cis and trans effects of the risk variants of coronary artery disease in the Chr9p21 region
Source: BMC Med Genomics. 2015 May 10;8:21. doi: 10.1186/s12920-015-0094-0 (PMC4432789; doi:10.1186/s12920-015-0094-0)
Supplement: Additional file 1: Table S1. — Four SNPs in the Chr9p21 region associated with all four top transcripts identified by SKAT_Beta(1,25). [file 12920_2015_94_MOESM1_ESM.pdf]

Supplemental Table 1. Four SNPs in the Chr9p21 region associated with all four top transcripts identified by SKAT_Beta(1,25)

SNP	Coded allele frequency	Coded allele	Non coded allele	P value from single SNP analysis			
				DUT (ENST00000331200)	EIF1AY (ENST00000361365)	CASP14 (ENST00000427043)	ABCA1 (ENST00000374736)
rs16923583	0.9944	T	A	2.14E-08	1.21E-05	1.95E-07	1.16E-06
rs16905597	0.9951	G	A	2.56E-08	3.86E-06	1.98E-06	1.65E-07
rs73650008	0.9951	T	C	2.75E-08	3.96E-06	2.01E-06	1.70E-07
rs41386451	0.9952	A	C	2.89E-08	3.66E-06	8.39E-07	1.82E-07
