# Supplementary figures and images for: The cis and trans effects of the risk variants of coronary artery disease in the Chr9p21 region
Source: BMC Med Genomics. 2015 May 10;8:21. doi: 10.1186/s12920-015-0094-0 (PMC4432789; doi:10.1186/s12920-015-0094-0)

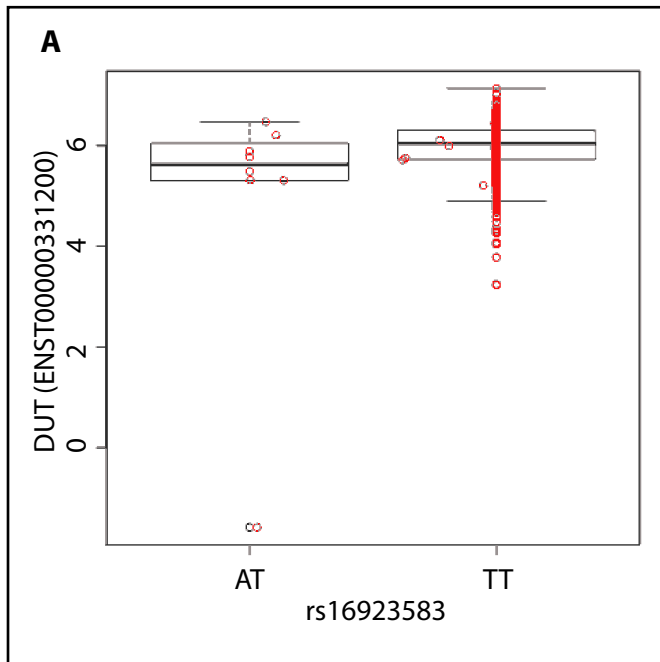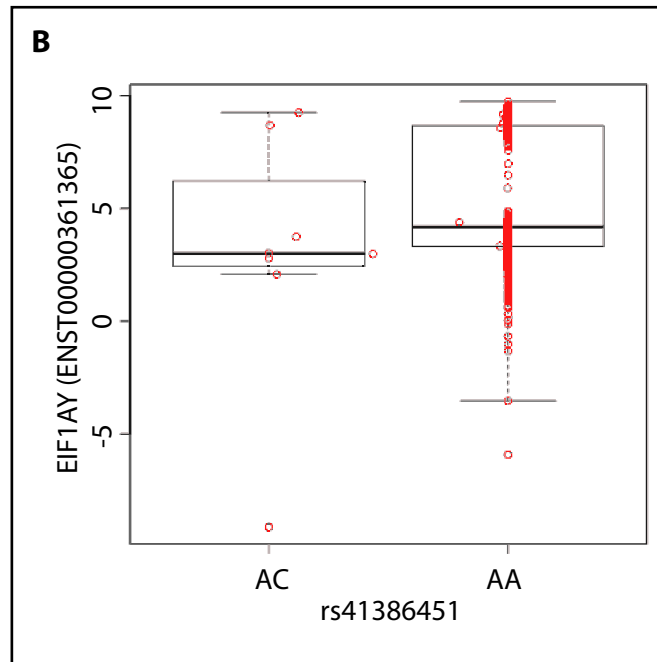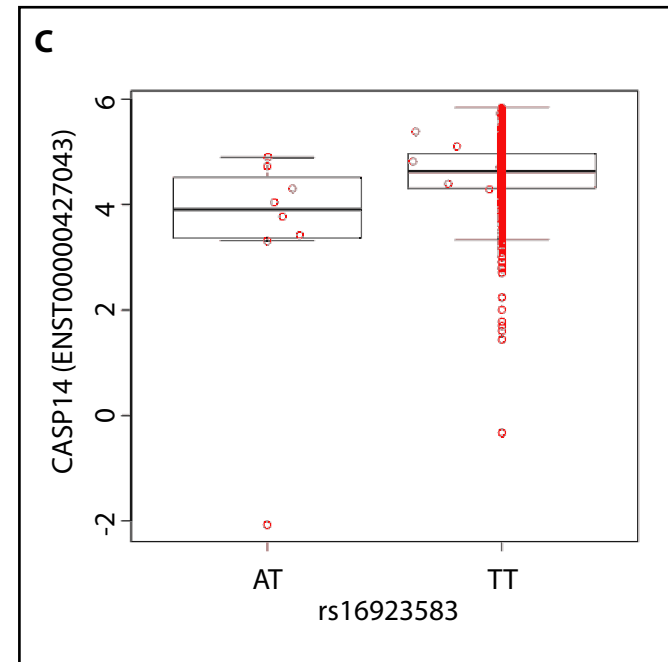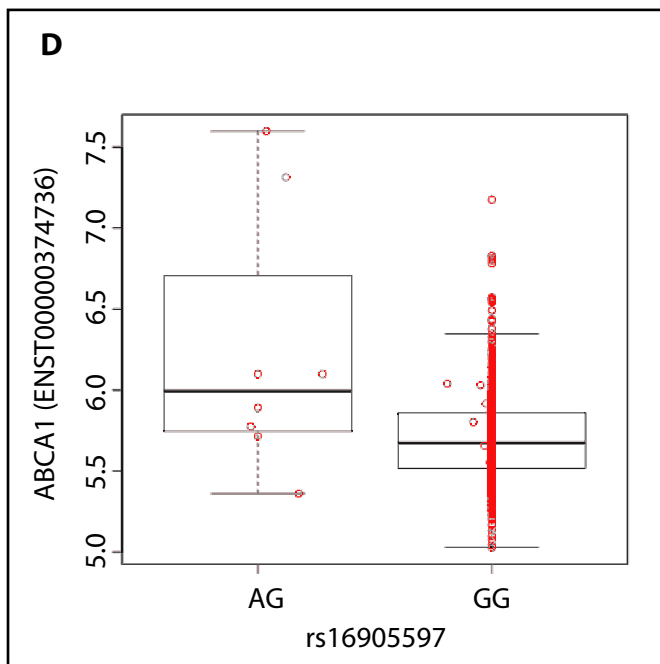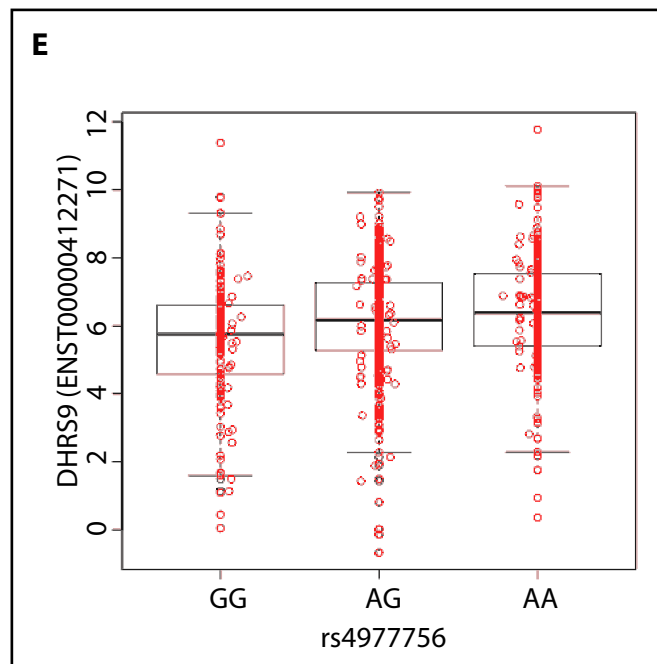

Supplement: Additional file 2: Figure S1. — The mRNA expression (log2 scale) of the top 5 trans-effected transcripts (identified by SKAT) was plotted against each genotypes for the corresponding top SNPs (shown in red). Boxplot was added on top of each scatterplot to show the median and interquartile range of the expression level for each genotypes (shown in black). The five plots are for gene A: DUT (ENST00000331200), B: EIF1AY (ENST00000361365), C: CASP14 (ENST00000427043), D: ABCA1 (ENST00000374736) and E: DHRS9 (ENST00000412271). [file 12920_2015_94_MOESM2_ESM.pdf]
